# Rediscovery of *Leptoxis compacta* (Anthony, 1854) (Gastropoda: Cerithioidea: Pleuroceridae)

**DOI:** 10.1371/journal.pone.0042499

**Published:** 2012-08-08

**Authors:** Nathan V. Whelan, Paul D. Johnson, Phil M. Harris

**Affiliations:** 1 Department of Biological Sciences, University of Alabama, Tuscaloosa, Alabama, United States of America; 2 Alabama Aquatic Biodiversity Center, Alabama Department of Conservation and Natural Resources, Marion, Alabama, United States of America; Smithsonian's National Zoological Park, United States of America

## Abstract

The Mobile River Basin is a hotspot of molluscan endemism, but anthropogenic activities have caused at least 47 molluscan extinctions, 37 of which were gastropods, in the last century. Nine of these suspected extinctions were in the freshwater gastropod genus *Leptoxis* (Cerithioidea: Pleuroceridae). *Leptoxis compacta*, a Cahaba River endemic, has not been collected for >70 years and was formally declared extinct in 2000. Such gastropod extinctions underscore the imperilment of freshwater resources and the current biodiversity crisis in the Mobile River Basin. During a May 2011 gastropod survey of the Cahaba River in central Alabama, USA, *L. compacta* was rediscovered. The identification of snails collected was confirmed through conchological comparisons to the *L. compacta* lectotype, museum records, and radulae morphology of historically collected *L. compacta*. Through observations of *L. compacta* in captivity, we document for the first time that the species lays eggs in short, single lines. *Leptoxis compacta* is restricted to a single location in the Cahaba River, and is highly susceptible to a single catastrophic extinction event. As such, the species deserves immediate conservation attention. Artificial propagation and reintroduction of *L. compacta* into its native range may be a viable recovery strategy to prevent extinction from a single perturbation event.

## Introduction

The Mobile River Basin (MRB) in Alabama and Georgia contains the highest levels of freshwater molluscan biodiversity in North America [1], [2], [3], [4]. Anthropogenic activities, however, have caused massive declines in gastropod biodiversity throughout the basin. At least 47 molluscan extinctions (37 gastropods) and many other local extirpations were the immediate result of inundation for hydropower, channelization for navigation and pollution from mine and urban centers throughout the mid 20^th^ century [3], [4], [5]. These extinctions comprise a third of known molluscan extinctions globally [6], making the MRB a major component of the global biodiversity crisis.

Freshwater gastropods in the family Pleuroceridae (Gastropoda: Cerithioidea), suffered the largest number of the aforementioned MRB extinctions (∼29) [4]. Of the 14 native MRB *Leptoxis* species, nine are considered extinct, including *L. compacta* [7]. Four of the remaining five *Leptoxis* are classified under the U.S. Endangered Species Act as either threatened or endangered [8], [9]. Remaining *Leptoxis* species in the MRB are of high conservation concern, and they are the focus of active propagation and reintroduction efforts [10], [11], [12].

*Leptoxis compacta* was formally declared extinct in 2000 [13], and was not collected in a 1992 survey for the US Fish and Wildlife Service (USFWS) [14], a 2005 ADCNR survey of the Cahaba River [15]or in a more recent survey by Tolley-Jordan [16]. It is the only pleurocerid endemic to the Cahaba River considered extinct [7], [13], and has not been collected in at least 70 years. Historically, *L. compacta* was most abundant in the central section of the Cahaba River at Lily Shoals in Bibb County, Alabama [17], [18]. Exact causes of *L. compacta*'s decline are unknown, but the species was declining in abundance and range by 1935 [17]. The snail's decline was likely a result of its naturally small range, pollution from mines in the area, and effluent from the Birmingham, Alabama metropolitan area [19].

In this study, we describe the results of targeted surveys for *L. compacta* in the middle portion of the Cahaba River in Bibb and Shelby Counties, Alabama. Whenever an “extinct” species is putatively re-discovered, special care must be taken to confirm the identity of the species [20]. As such, the conchological and radular morphology of *L. compacta* individuals collected in this study are compared to historically collected material along with other sympatric pleurocerids to confirm the identity and distinctness of *L. compacta*. We also document for the first time, the egg-laying strategy, juvenile morphology, and soft tissue pigmentation of *L. compacta*. Potential threats to the long-term survival of *L. compacta* are also discussed.

## Results

### Survey

**Figure 1:**
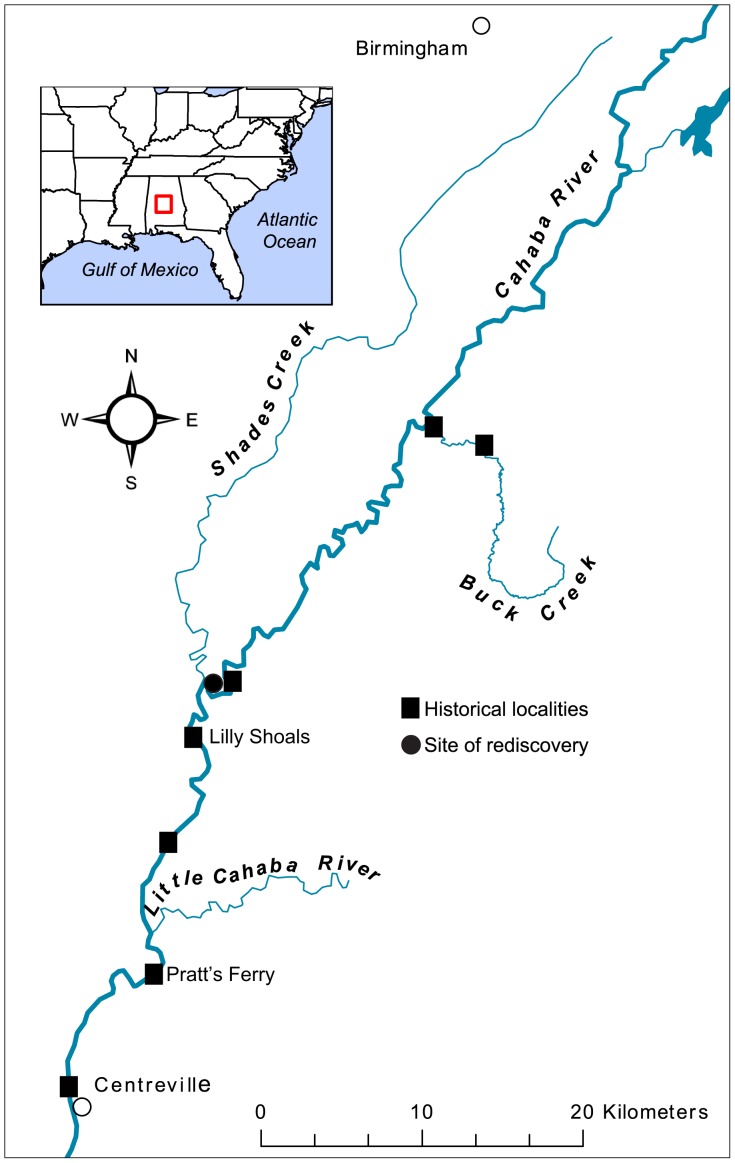
Map of the Cahaba River and select tributaries. Historical collections sites were sampled in August 2011, but *L. compacta* was found only at the site of re-discovery.

Museum lots of *L. compacta* reviewed are listed in Table 1 and historical localities are labeled on Figure 1. The historical range of *L. compacta* extended from Centerville, Bibb County, Alabama, USA upriver and into lower Buck Creek in the Valley and Ridge physiogeographic province of the southern Appalachian Mountains. The most recent lots we analyzed were from 1933 (UMMZ 57871, MCZ 98217), and as far as we are aware this was the last time the species was collected.

**Table 1:** Locality and museum catalogue numbers for *L. compacta* museum lots analyzed in this study.

Locality	Catalogue #
Alabama (river unspecified), Lectotype	MCZ 072063
Buck Creek	FLMNH 81147; USNM 158595
Cahaba River (location unspecified)	USNM 15874, 218694, 158741, 509539*, 158743, 407631; UMMZ 55741
Cahaba River at Abita	USNM 321957
Cahaba River at Centerville	UMMZ 57871
Cahaba River at Lily Shoals	FLMNH 81172, 81188, 81144, 7136; USNM 590380
Cahaba River near Piper	FLMNH 81137, 81178
Cahaba River at Pratt's Ferry	FLMNH 81165, MCZ 98217

* Lot from which radulae were taken.

*Leptoxis compacta* was found during the May 2011 survey on an unnamed shoal upstream of the Cahaba River and Shades Creek confluence in Shelby County Alabama (Fig. 1; 33.1786°N, 87.0174°W). At this site, we found every pleurocerid species known from the middle Cahaba River including the federally threatened Round Rocksnail, *L. ampla* (Anthony 1855) (Table 2) [17], [21]. We also found the endemic limpet *Rhodacmea cahawbensis* Walker, 1917 (Planorbidae) and *Lepyrium showalteri* (Lea, 1861) (Lithoglyphidae), which is federally endangered.

**Table 2:** Species collected in the May 2011 survey at the site of *L. compacta* re-discovery (33.178601°N, 87.017481°W).

Species	Catalogue #
*Elimia annettae*	USNM 1186562
*Elimia ampla*	USNM 1186561
*Elimia cahawbensis*	USNM 1186563
*Elimia clara*	USNM 1186564
*Elimia showalteri*	USNM 1186566
*Elimia variata*	USNM 1186567
*Leptoxis ampla*	USNM 1186568
*Leptoxis compacta*	USNM 1186565 FLMNH 449320
*Pleurocera prasinatum*	USNM 1186569
*Rhodacmea cahabensis*	USNM 1186570

*Lepyrium showalteri* was found, but not collected due its endangered status under the U.S. Endangered Species Act.

During the first survey we failed to locate *L. compacta* below the Shades Creek confluence. Furthermore, the second survey for *L. compacta* (Fig. 1) also failed to locate the species from other historical sites. At every site *L. compacta* was historically found that we sampled, other pleurocerid species were present.

### Life history and morphology

**Figure 2:**
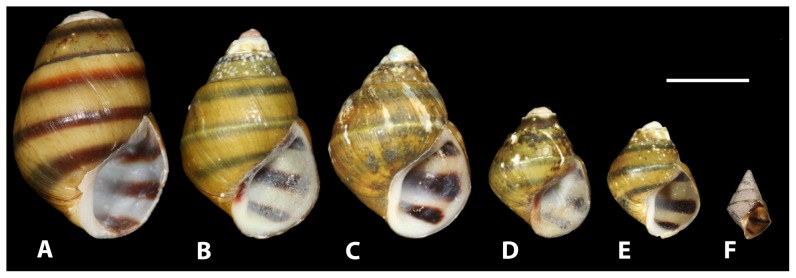
Growth series of *L. compacta*. A) *L. compacta* lectotype (MCZ 072063). B–E) Wild caught individuals. F) Juvenile grown in culture, approximately 4 months old. Scale bar = 5 mm.

**Figure 3:**
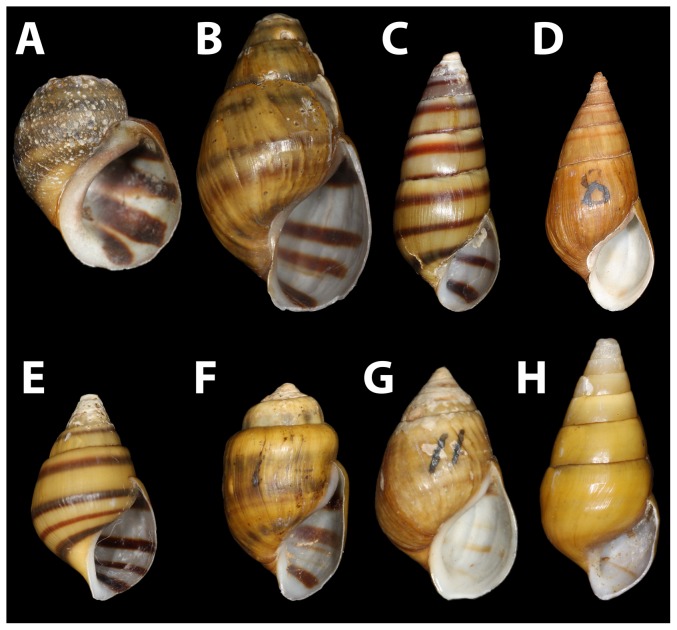
Lectotypes of pleurocerids sympatric with *L. compacta*. A) *L. ampla* (MCZ 161803). B) *E. ampla* (MCZ 161735). C) *E. annetae* (UMMZ 128908). D) *E. cahawbensis* (USNM 119055). E) *E. clara* (MCZ 072329) F) *E. showalteri* (ANSP 26881) G) *E. variata* (USNM 118756). F) *P. prasinatum* (USNM 122206).

**Figure 4:**
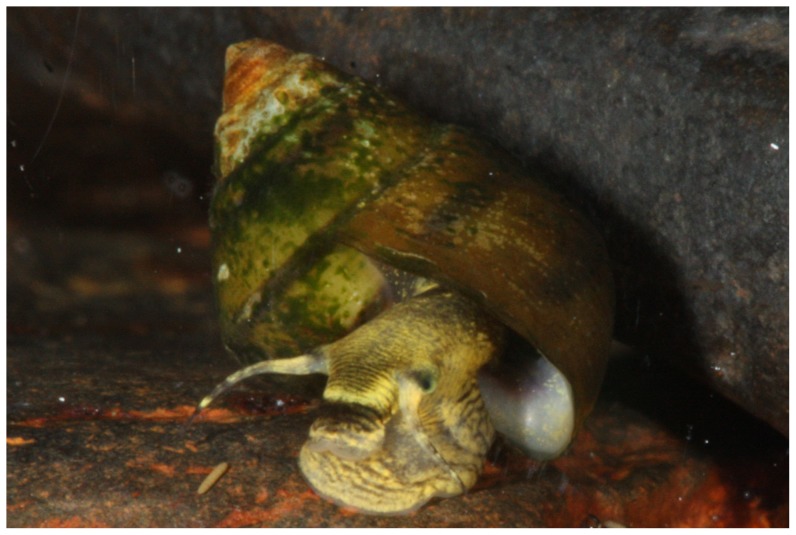
Photograph of live *L. compacta* from the Cahaba River, Shelby County, Alabama.

**Figure 5:**
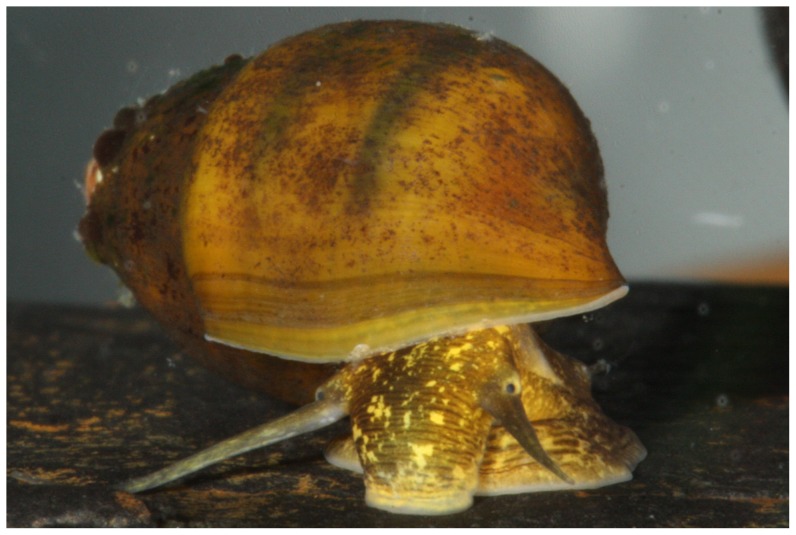
Photograph of live *E. clara* from the Cahaba River, Shelby County, Alabama.

Snails we collected and putatively identified as *L. compacta* possess shells nearly identical to the lectotype (Fig. 2) and the original species description [22]. *Leptoxis compacta* shells do not closely resemble those of other sympatric species (Figs. 2, 3). Juvenile *L. compacta* shells possess one distinct carina on the main body whorl, which is eventually lost as adults (Fig. 2). Individuals with shell pigmentation lines are always present in three wide bands. Most wild-caught individuals had purple pigmentation on the columella indentation, but this trait was not observed in juveniles propagated in captivity. The external tissue pigmentation of *L. compacta* is yellow, mottled with black and includes prominent black bands in the middle of the proboscis and on both eyes (Fig. 4). This pigmentation banding pattern is identical to sympatric *L. ampla* (not figured). Pigmentation patterns and the presence of an ocular peduncle are features that distinguish *L. compacta* from sympatric *Elimia* spp. including *E. clara* (Anthony, 1854) (Fig. 5), which is conchologically most similar to *L. compacta* (Figs. 2, 3).

**Figure 6:**
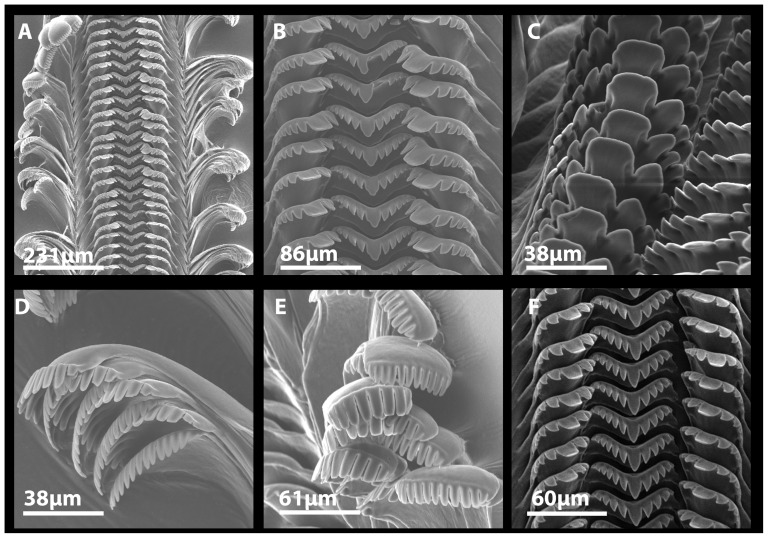
Scanning electron micrographs of *L. compacta* radulae collected in May 2011 (A–E) and the radula of historically collected individual (F). A) Section of anterior radular ribbon. B) Detailed view of rachidian and lateral teeth. C) View of lateral teeth at 45 degree angle, and slightly rotated counter-clockwise. D–E) Views of marginal teeth showing variation between individuals. F) Rachidian and lateral teeth removed from individual collected in 1881 (USNM 509539).

The radular structure of *L. compacta* specimens collected in May 2011 is identical to that of individuals collected in 1881 (Fig. 6). The basal margin of the rachidian tooth is widely convex. The central cusp is blunt and flanked by 4–5 denticles, with the outermost being weakly developed in most cases (Fig. 6 A, B). The lateral tooth contains one larger rectangular central cusp that is flanked by 4–5 outer denticles and 3–4 inner denticles (Fig. 6 B, C). The inner marginal teeth contain 10–12 denticles (Fig. 6 D, E). The number of denticles on the outer marginal teeth varies from 12–16, within and among individuals, and the outer denticles are often weakly developed (Fig. 6D, E).

**Figure 7:**
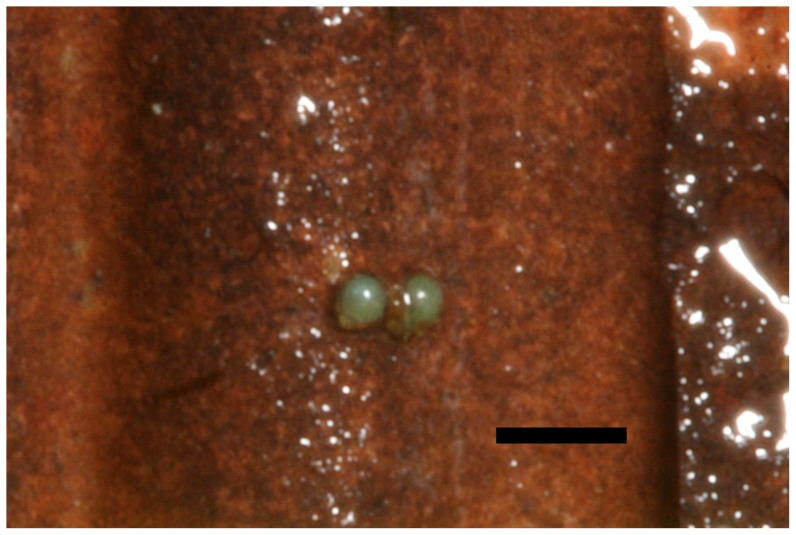
Photograph of two eggs that were laid by captive *L. compacta.* Scale bar = 1 mm.

Eggs were laid by female *L. compacta* within three days of being transferred into captive culture. This suggests female snails were laying eggs in the wild when collected in May 2011. Oviposition ceased when the daily maximum water temperature reached 29°C. Eggs were laid either singly or in a linear sequence (Fig. 7). Each egg was approximately 0.3 mm in diameter. Average length of the line of eggs was 1.57 eggs (n = 51 egg lines) with a maximum observed length of 3 eggs.

## Discussion

Only two types of morphological data are available to confirm the putative re-discovery of *L. compacta*: shell and radular. The primary type, shell descriptions in taxonomic works [18], [22] and museum records match the *L. compacta* we collected. Furthermore, radulae we extracted from *L. compacta* collected in 1881 are identical to those of live *L. compacta* collected in this study (Figure 6). Compared to sympatric pleurocerids, *L. compacta* shells most resemble those of Cahaba River *Elimia* spp. (Figs. 2, 3). Furthermore, the radular morphology is more similar to those of sympatric *Elimia* spp. than *L. ampla* [23], [24]. However, body pigmentation of *L. compacta* is most similar to that of *L. ampla* rather than sympatric *Elimia* (Figs. 4, 5). The aforementioned features of *L. compacta* are similar in some regards to both sympatric *Elimia* spp. and *L. ampla*, but taken in total distinguish *L. compacta* as unique. Molecular systematic analyses are underway to clarify the genetic position of *L. compacta*.

The reduced range of *L. compacta* qualifies the species as critically endangered under International Union for the Conservation of Nature criteria [25]. It is unclear why *L. compacta* suffered such a drastic range reduction while other sympatric pleurocerid species did not [15], [17]. However, point-source pollution and urban runoff from the Birmingham, Alabama metropolitan area threaten the long-term survival of *L. compacta*. Furthermore, the lone population of *L. compacta* is found adjacent to a large youth camp currently under construction. The U.S. Fish and Wildlife Service should consider *L. compacta* for protection under the U.S. Endangered Species Act because of its highly restricted range and susceptibility to a single pollution or siltation event. The ADCNR has already undertaken culturing projects for federally endangered *L. plicata* and *L. foremani* to expand their current range [10], [11], [12], and we argue that similar efforts should be pursued for *L. compacta*.

There is little literature on the reintroduction of freshwater gastropods [26], but general conservation rules [25], [27] and guidelines for reintroduction of fish are applicable for freshwater snails [28], [29], [30]. There are two sites within the historical range of *L. compacta* that we consider potentially viable for the reestablishment of a second population. Lily Shoals, which is isolated from development or bridge crossings, is 5.8KM downstream of the remaining *L. compacta* population and supports at least five other species of pleurocerids [17]. The second potential site for reintroduction is 25.8KM downstream of the site of rediscovery at Pratt's Ferry. Downstream sites are ideal for establishing a second *L. compacta* population because pleurocerid snails have a net upstream movement, thus have potential to naturally colonize upstream localities [31], [32]. These localities are also better suited than upstream sites because primary production is generally higher downstream [33]. Furthermore, the lone tributary that historically harbored *L. compacta*, Buck Creek, has three wastewater discharge points and suboptimal habitat [34], [35]. Extensive assessments should be performed to identify additional sites for reintroduction that will enhance the survival prospects of propagated *L. compacta* [11], [36].

Through comparisons to the *L. compacta* lectotype, and radulae from fresh and historic collections we present compelling evidence that *L. compacta* has been “re-discovered” in the Cahaba River in Shelby County, Alabama. Why three previous surveys of the Cahaba River—including the site of re-discovery—failed to locate *L. compacta* is unknown [14], [15], [16]. Because of its restricted range, *L. compacta* should be the focus of immediate conservation attention. Nevertheless, the rediscovery of *L. compacta* is an encouraging moment in the recent history of conservation and biodiversity studies of freshwater mollusks in the MRB.

## Materials and Methods

### Survey

Alabama Department of Conservation and Natural Resources scientific collecting permits and U.S. Fish and Wildlife Service permits for threatened species were obtained prior to sampling. Since *L. compacta* does not have a formal status under the U.S. Endangered Species Act, federal permit authorization does not apply.

To document the historical range of *L. compacta* and the approximate last collection of the species, museum specimens were analyzed at the National Museum of Natural History (NMNH), North Carolina Museum of Natural Sciences (NCMNS), and the Florida Museum of Natural History (FLMNH). Photographs of *L. compacta* lots housed at the Museum of Comparative Zoology at Harvard University (MCZ) and the University of Michigan Museum of Zoology (UMMZ) were also examined (Table 1). Spurious localities represented by only one lot and outside the otherwise documented range of *L. compacta* (*e.g.* “Alabama River at Selma,” USNM 178542) were excluded from consideration.

In May 2011 gastropods were qualitatively surveyed from a kayak in the Cahaba River (Fig. 1) from sites upstream of Shades Creek and Cahaba River confluence to below Piper Bridge. All other sites where *L. compacta* (Fig. 1) was historically found were surveyed in August 2011 to confirm range contraction of *L. compacta*. Live snails collected in May 2011 were transported to the Alabama Aquatic Biodiversity Center in Marion, Alabama for species identifications and preservation. Endangered species that we encountered were not collected. Identification of each species was based on comparison with primary types (Fig. 2), museum records, and descriptive literature [18], [21], [22], [37]. Snails collected in these surveys were preserved following Fukuda et al. [38] and deposited at NMNH and FLMNH (Table 2).

### Morphological and life history analyses

A size range of *L. compacta* individuals was collected from the site of re-discovery, and live *L. compacta* were photographed in an aquarium and compared to other sympatric pleurocerids. We extracted radulae from two *L. compacta* specimens collected in the original May 2011 survey and from two samples of dried tissue left in shells from individuals collected in 1881 (USNM 509539). Radulae were extracted following the procedure of Holznagel [39]. *Leptoxis compacta* radulae were visualized on a Hitachi S-2599 Scanning Electron Microscope at the University of Alabama Optical Analysis Facility.

Approximately thirty *L. compacta* individuals were placed in captivity at the Alabama Aquatic Biodiversity Center to observe egg-laying behavior. Because of the difficulties of recording egg-laying strategies in the wild, a culturing environment is ideal for these observations [40]. Snails were kept in a 20 L acrylic tank with a 0.83 cm bulkhead fitting that allowed a constant exchange of thermally ambient well water. A 15 L/min powerhead was attached to the lid of the tank to create a constant flow regime. An airstone was placed in the tank to saturate the water with dissolved oxygen. Water temperature was measured hourly with a Hobo Temp Logger (Onset Computer Corporation).

At least every three days, tanks were checked for eggs and the eggs were counted and the adjacent area of the tank marked with a permanent marker to insure individual eggs were not counted twice. Juveniles were allowed to grow in the culturing environment for 5.5 months. Growth series of wild caught and cultured snails were compared to demonstrate conchological changes during ontogeny.
